# Effect of Iso-Caloric Substitution of Animal Protein for Other Macro Nutrients on Risk of Overall, Cardiovascular and Cancer Mortality: Prospective Evaluation in EPIC-Heidelberg Cohort and Systematic Review

**DOI:** 10.3390/nu15030794

**Published:** 2023-02-03

**Authors:** Rashmita Bajracharya, Verena Katzke, Trasias Mukama, Rudolf Kaaks

**Affiliations:** Department of Cancer Epidemiology, German Cancer Research Center (DKFZ), 69120 Heidelberg, Germany

**Keywords:** animal protein, cardiovascular mortality, cancer mortality, macronutrients

## Abstract

Ecological studies showed correlations between a shift toward animal-protein-rich diets and longer life-expectancy; however, only a few studies examined individual-level association of protein source and mortality risks using appropriate iso-caloric substitution models adjusted for total energy intake. We used EPIC-Heidelberg (European Prospective Investigation into Cancer and nutrition) to create iso-caloric substitution models and determined relative all-cause, cardiovascular and cancer mortality hazards associated with dietary intake of animal protein and other macronutrients, employing Cox proportional hazard models. For comparison with other studies, we also synthesized evidence from a systematic review relating animal protein intake to mortality risk from seven prospective cohort studies in the USA, Europe and Japan. Substitution of 3% of total energy from animal protein for fat (saturated, mono-unsaturated) and carbohydrate (simple, complex) was associated with all-cause mortality (Hazard Ratios [HR] from 1.05 to 1.11), mostly driven by cardiovascular mortality (HR from 1.13 to 1.15). Independently of animal protein, substituting poly-unsaturated fat for saturated fat increased cancer-related mortality risk by 12 percent. The systematic review largely corroborated our findings. Overall, higher proportions of dietary energy from animal protein, combined with low energy intake from either carbohydrate sub-types or dietary fats, increases all-cause and cardiovascular mortality risks, but not cancer-related mortality.

## 1. Introduction

A long-standing question in nutrition epidemiology concerns the optimal dietary macronutrient composition in relation to an individual’s overall health. On a population level, national statistics of food availability have documented important changes in average per capita availability and consumption of food types along with the economic development of countries [1]. Overall, these changes are related to major shifts in the average macronutrient composition of diet. One of these is a shift towards a higher percent of energy intake in the form of animal protein, compensated by a lower percent of energy from vegetable protein, in higher- (e.g., Europe, North America, Australia) compared with lower-income countries (e.g., countries in South Asia and Africa). In parallel, the average diet composition in higher-income countries is also characterized by higher percentages of dietary energy intake from total and saturated fats, as well as from added sugar and other refined carbohydrates [2,3]. Within lower- and middle-income countries, similar shifts in diet composition can be observed when comparing individuals living in urban, as opposed to more rural, environments [1]. 

Between-country comparisons have shown strong positive correlations between the average per capita availability of animal protein, sugar and total and saturated fats and incidence rates of cancer, diabetes and cardiovascular incidents [4]. By contrast, inverse correlations are observed with rates of all-cause mortality, as the overall life-expectancy is generally higher in the higher income countries [3]. These contrasting observations raise questions as to whether or not ecological correlations of diet with disease and mortality risks, as observed across countries, reflect similar associations between diet, disease and mortality on the level of single individuals, and whether associations reflect any causal mechanisms. 

To assess associations on an individual level, several recent studies have addressed the relationship between macronutrient composition of diet and all-cause and cause-specific mortality rates in prospective cohorts in the USA, Australia, Japan and several European countries, focusing analyses on energy intake in the form of animal protein as compared with energy from plant protein or other macronutrient sources [5,6,7,8,9,10]. Most of these linked higher intake of animal protein, or lower intakes of plant protein, with increased overall or cardiovascular mortality rates, or both [6,7,8,9,10]. To be fully interpretable, it is important that these associations are examined with adjustment for total energy intake, and that risk effects are estimated for specific iso-caloric substitutions of one macronutrient for another. In several studies so far, however, estimates were derived from statistical models that used different degrees of macro-nutrient breakdown (i.e., for sub-types of dietary protein [animal or plant], fats [saturated, mono-unsaturated or poly-unsaturated] and carbohydrates) and considered variable substitution effects. Thus, mortality risks have mostly been estimated in association with the substitution of animal protein intake either for carbohydrate [6,9] or for plant protein [11], but did not generally examine further effects that could have been calculated from the same risk models. 

Here, we report findings from a prospective EPIC-Heidelberg cohort, using statistical models to comprehensively examine relative mortality risks (all-cause, cardiovascular and cancer mortality) associated with different possible iso-caloric substitutions of animal protein for other possible macronutrient sources, as well as for further possible nutrient substitutions independently of animal protein. In addition, we performed a systematic review of previous iso-caloric modeling studies on the effect of animal protein substitutions, so as to compare their findings with those from our own analyses, and to generate an overall synthesis of evidence relating animal protein intake to mortality risk from prospective studies so far.

## 2. Materials and Methods

### 2.1. Study Population: The EPIC-Heidelberg Cohort

EPIC-Heidelberg recruited participants and collected data between 1994 and 1998 as part of the larger European EPIC study. The study design and methods for the European EPIC study, including the Heidelberg component, have been described previously [12,13]. Briefly, EPIC is a multi-center prospective cohort study designed to investigate nutrition and cancer associations with a potential to explore further relations with other diseases and mortality. EPIC enrolled 519,978 participants in 23 centers located in 10 European countries. The EPIC-Heidelberg cohort included 25,540 study participants aged 35–65 years recruited from the general population living in the southern German city of Heidelberg and its surrounding municipality at recruitment [14]. Baseline examinations included a detailed medical interview and comprehensive questionnaire assessments of socioeconomic status, lifestyle factors and habitual diet. Anthropometric measurements were taken by trained personnel and blood samples were obtained from 95% of the participants. Informed consent was obtained from all participants at baseline. 

### 2.2. Assessment of Habitual Diet

Information about habitual diet was collected using a self-administered food frequency questionnaire (FFQ), which had been extensively validated in prior studies [15,16,17]. Briefly, a total of 158 single foods or mixed dishes were included. For each food item the participant provided information about the consumption of the food in the last year, frequency of consumption (1–6 times) and the time period (day, week, month or year). A semi-quantitative questionnaire was used, requesting information not only about the frequency of consumption but, for a number of food items, also about habitual portion sizes. To increase the accuracy of portion size estimation, photographs of food portions of various sizes were included. The estimation of total energy and macro nutrient intake based on the FFQ was also validated [15]. A food composition database [18] was used to convert food consumption data into estimated intakes of nutrients and total energy, and estimated intakes of total energy and macronutrient intakes were also validated through comparison with intake estimates assessed by a different method (repeat 24 h recalls) [15,16,17]. 

Total energy intake was defined as the daily energy intake from total protein, total fatty acids and total carbohydrates. Since alcohol is not considered feasible with regard to nutrient substitution, alcohol was not included in total energy intake but was adjusted in the models as a covariate [2,19]. The data compiled on the EPIC nutrient database only gave qualitative information about the source of food, and the source was classified based on the predominant origin of the food (animal/plant) [20]. Dietary total protein was composed of animal protein (100% or above 95% animal origin), plant protein (100% or above 95% plant origin) and ‘unknown’ origin. Unknown includes the protein where the origin (either animal or plant) was not clear, such as with protein-based artificial sweeteners or chewing gums. Unknown sources of protein contributed to 2.6% of total energy and, for this study, ‘unknown’ was combined with plant protein sources and from here forth will be referred to as non-animal protein. Dietary total fat was composed of saturated fatty acid, mono-unsaturated fatty acid and poly-unsaturated fatty acid. Because of the lack of information and standardization of the nutrients across national food composition databases, a portion of total fat energy was left unclassified in the EPIC database. Therefore, the unclassified fat that contributed to 2.4% of total energy intake was not included in the analysis to prevent misclassification. Total carbohydrate was composed of mono- and di- saccharides and other carbohydrates (oligosaccharides and polysaccharides).

### 2.3. Prospective Ascertainment of Mortality Endpoints

In EPIC-Heidelberg, mortality outcomes were ascertained first through regular record linkages with municipal registries for vital status, and for all cases of death further information on causes of death (death certificates) was collected from regional health offices, which was coded according to the International Statistical Classification of Diseases and Related Health Problems (ICD)-10 by trained medical study personnel. The present analyses are based on complete case ascertainments from June 1994 to May 2019. 

### 2.4. Statistical Analyses

After the exclusion of participants with prevalent cancer diagnosis (*n* = 949) at recruitment and those in extreme 1 percentile of ‘energy intake/energy requirement’ ratio (*n* = 485), 24,106 remained for the analysis. Those with unknown information about smoking history (*n* = 341) were imputed using a fully conditional specification multiple imputation method [21]. Relative mortality hazards (hazard ratios [HR]) and 95% confidence intervals (CI) were estimated using Cox proportional hazard models and cause-specific Cox models, with age as the underlying time scale, to determine the association of mortality hazards with dietary macro-nutrient composition. To determine macro-nutrient breakdown for the final model, several models with different degrees of macro-nutrient breakdown were compared using a likelihood ratio test. Further detail on the step-wise macro-nutrient breakdown along with their corresponding deviance (−2 logL), chi-square and *p*-value are provided in Table S1. Models were built to estimate the predicted changes in mortality risk associated with an iso-caloric substitution of 3% of total energy intake from one macronutrient for any of the other macronutrients in the model, using a nutrient density model (ND) [22] adjusted for age, sex and total energy intake (in kcal/day) as the potential confounding variables. The ND model included model terms for percent energy from animal protein, non-animal protein, saturated fat, mono-unsaturated fat, poly-unsaturated fat, mono- and di-saccharides and other carbohydrates. Leave-one-out method was used such that the macronutrient being substituted was always left out of the ND model [23]. To test for further potential confounding, model variants were generated that stepwise included additional covariates for smoking (never, long-time quitter, short-time quitter, current light, current heavy and pipe/cigar/occasional), body mass index (BMI) (kg/m^2^), intake of alcohol (non-drinkers, 0–6 g/day, 6–12 g/day, 12–24 g/day, 24–60 g/day, 60–96 g/day and >96 g/day) and dietary fiber (g/day). Proportional hazard assumption in the Cox model was tested using Schoenfeld residuals [24]. Statistical significance was defined as *p* < 0.05 or 95% confidence intervals excluding the null, and all analyses were performed using SAS Version 9.4 (SAS Institute, Inc., Cary, NC, USA). 

To examine the robustness of ND models, we additionally performed an equivalent series of analyses using “standard” linear models (“S” models) [22], in which intakes of macronutrients were modeled as absolute intakes expressed in kcal/day, in addition to total energy intake. Since participants with diabetes diagnoses could have received specific diet recommendations, which could impact dietary pattern and influence the outcome, a sensitivity analysis was performed by excluding those with a diabetes diagnosis at the time of recruitment (*n* = 829) from the analytic sample. Many past studies have also suggested that the mortality association with animal protein may be largely driven by red meat consumption [10,25]. To examine this further we additionally performed models with total red meat intake (in g/day) as an adjustment factor. 

### 2.5. Method for the Systematic Review

**Figure 1:**
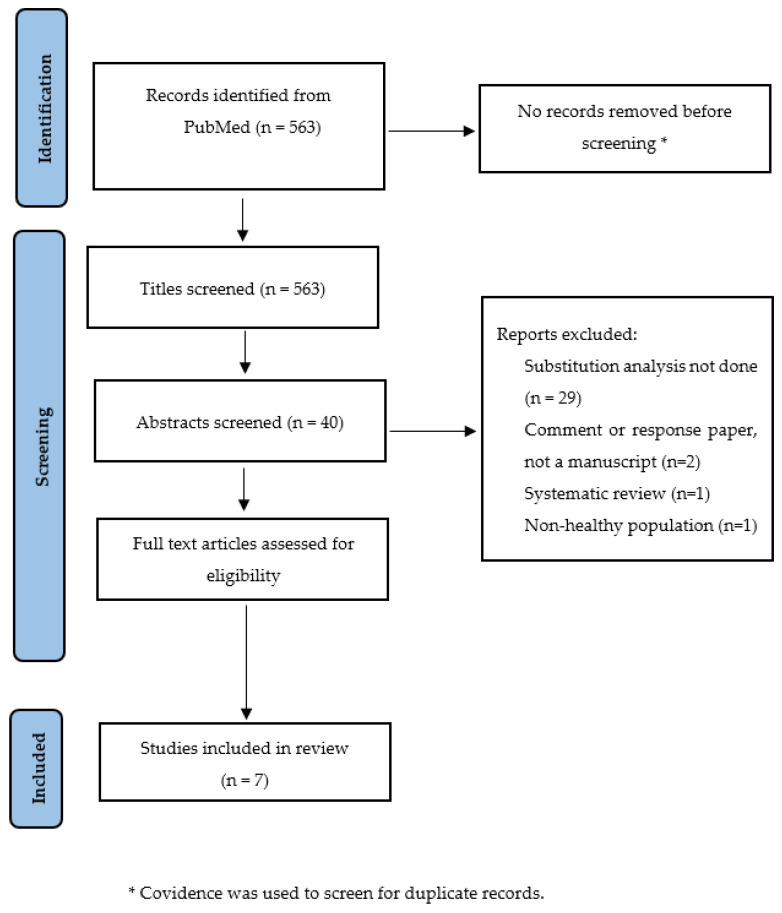
PRISMA flow diagram showing search strategy and selection process.

The systematic review was conducted and reported based on pre-defined PRISMA guidelines [26] and was registered in PROSPERO (ID = CRD42022384668). A Pubmed search was conducted using the search terms provided in Table S2. Two reviewers screened all titles or abstracts. The studies were included for final review if (1) the study design was a prospective cohort study, (2) it included healthy sample at baseline, (3) an isocaloric substitution analysis was performed using either ND model or S model and (4) it reported the risk estimates for the association of animal protein intake and mortality. Systematic reviews/meta-analyses were not included. However, additional manuscripts were searched by reviewing the reference list of prior systematic reviews/meta-analyses. A PRISMA diagram for the search strategy and study selection process is provided in Figure 1. The following information were extracted from the eligible studies: first author, year of publication, model implemented (ND or S), nutrient/food breakdown, sample size, duration of follow-up, number of deaths reported (all-cause, cardiovascular and cancer), covariates adjusted in the models, name of the study cohort (where applicable), country where the study was conducted, unit used for the interpretation of the result and estimated hazard ratios. The Joanna Briggs Institute (JBI) critical appraisal tool was used to appraise the quality of the retrieved studies [27]. 

## 3. Results

### 3.1. Results in EPIC-Heidelberg Cohort

#### 3.1.1. Cohort Characteristics

Among the 24,106 participants retained for the present analyses, a total of 4029 cases of death were registered until the end of the follow-up (May 2019), of whom 982 (24.3%) died of cardiovascular events, 1603 (39.7%) of cancer and the remaining 1444 (35.8%) of other, miscellaneous conditions (Table 1). The median age of the participants at recruitment was 51.4 (Inter-Quartile Range [IQR] = 43.5–57.5) years. Fifty-three percent were female and a majority (42.7%) of the participants had a BMI ranging from 18 to 24 kg/m2. More than 40% of the participants reported that they never smoked and 5% of the participants were non-drinkers. About 11% of the participants reported being current heavy smokers. The total energy intake of the overall sample was 1973.6 kcal with 152.1 kcal contributed by energy from animal protein and 139.6 kcal contributed by energy from non-animal protein. The duration of follow-up for those who died was 16.1 (IQR= 10.6–22.5) years, and that for those still alive in May 2019 was 22.9 (IQR = 22.1–23.9) years. 

#### 3.1.2. Model Selection

The model with maximum macro-nutrient breakdown (broken down into animal protein, non-animal protein, saturated fat, mono-unsaturated fat, poly-unsaturated fat, mono- and di-saccharides and other carbohydrates) was chosen for the final analysis as it had a significantly better model fit compared with models in which macronutrients were considered on a more aggregate level (Table S1). Using this model (Model 5, see Table S1), a total of 21 pairwise macro-nutrient substitution effects can be calculated. 

#### 3.1.3. Association of Animal Protein Intake with Mortality

A basic nutrient density model (ND) minimally adjusted only for age, sex and total energy intake as confounding variables (Table 2, Model A) showed that substituting animal protein for any of the other macro-nutrients was associated with a higher mortality risk (HR from 1.07 to 1.18 for substitution of 3% of total energy). When models were further adjusted for smoking and BMI (Table 2, Model B), and then additionally adjusted for alcohol consumption and fiber intake (Table 2, Model C), the estimated mortality risks for higher animal protein intake were progressively attenuated, but remained significant for the substitution of animal protein for saturated or mono-unsaturated fats (for substitution of 3% of total energy, HR = 1.09 [CI = 1.03–1.15] and HR = 1.11 [CI = 1.02–1.21], respectively), for mono- and di-saccharides (HR = 1.06 [CI = 1.03–1.11]) or complex carbohydrates (HR = 1.05 [CI = 1.01–1.09]). Independently of animal protein, the most extensively adjusted model (Model C) also indicated increased mortality risks in diets higher in poly-unsaturated fats, and correspondingly lower in either saturated fats (HR = 1.10 [1.04–1.17]) or simple (HR = 1.08 [CI = 1.01–1.14]) or complex carbohydrates (HR = 1.06 [CI = 1.004–1.13]). Looking at cause-specific mortality, estimated associations of animal protein intake with mortality appeared to be largely driven by increased mortality, especially due to cardiovascular causes (HR from 1.13 to 1.15, Table S3, Model C), whereas the mortality risks associated with higher poly-unsaturated fat intake (substitution of poly-unsaturated for saturated fats) appeared to be more specific for cancer deaths (Table S4). 

Excluding participants with diabetes diagnoses at the baseline and adjusting for red meat intake in the multivariate adjusted models did not significantly change the various associations observed between nutrient substitutions and mortality risk (Tables S5 and S6). In addition, the results from a complementary series of standard ‘S’ models were generally equivalent to those obtained by the ND models (Table S7). 

### 3.2. Systematic Review Result of Previous Prospective Cohort Studies

#### 3.2.1. Literature Search and Study Characteristics

Overall, 563 studies were identified in the initial search. As there were no duplicates, all 563 studies were included for title screening. Forty studies were included for abstract screening and seven studies were included for a full text review. All seven studies were included in the final review based on inclusion criteria. Table 3 shows the summary of study characteristics and findings in alphabetical order of first author’s last name. Four out of seven studies were conducted in the US, two in the Netherlands and one in Japan. All seven studies were prospective cohort studies; six used nutrient density ‘ND’ model and one used standard ‘S’ model, adjusting for total energy intake, and all seven studies provided estimates for the relative risk of mortality associated with increases in animal protein intake or animal-protein-rich foods such as red meat. The median follow-up of mortality as the end-point in the reviewed studies ranged from 13 years to 26 years.

#### 3.2.2. Association of Animal Protein Intake with Mortality

Most studies adapted a similar breakdown of macronutrients into components of proteins (animal and plant protein) and fats (saturated fat, mono-unsaturated fat, poly-unsaturated fat and trans/other fat), except for further breakdown into alcohol energy by Chen et al. (Table 3). Only three types of iso-caloric substitutions have been tested in the reviewed studies such that three studies examined substituting animal or plant protein for carbohydrate [6,9,25] and one study substituted plant protein for animal protein [11]. All three types of substitution scenarios linked higher animal protein intake (Chen et al., HR = 1.19, 95% CI = 1.04–1.37; Song et al., HR = 1.08 [CI = 1.01–1.16]), or lower plant protein intake (Kelemen et al., HR = 0.70 [CI = 0.51–0.98]; Sun et al., HR = 0.78 [CI = 0.70–0.87]), with higher cardiovascular mortality risk. However, all-cause mortality risk was higher when substituting animal protein for carbohydrate only in minimally adjusted iso-caloric models; the associations became statistically insignificant in Song et al. and Kelemen et al. after adjusting for further covariates. The adjustment of covariates only slightly attenuated the associations observed in other studies. Furthermore, in the included studies, the covariates adjusted in the maximally adjusted models varied, suggesting various degree of residual confounding, but each of the studies included important known confounders of diet intake and mortality association, such as smoking history, BMI, alcohol consumption and fiber intake. No studies found any association of animal protein intake and cancer mortality.

## 4. Discussion

In this analysis of the prospective EPIC-Heidelberg cohort, we found that animal protein stood out as an important predictor of all-cause mortality, in large part driven by the association of higher animal protein intake with increased risk of cardiovascular mortality. These increased risks were observed in relation to iso-caloric substitutions of animal protein either for saturated fats or for simple or more complex carbohydrates. Independently of animal protein, our analyses also indicated an increase especially in cancer-related mortality risk in association with higher intakes of poly-unsaturated fats, notably as iso-caloric substitution for saturated fats. 

Our findings of increased cardiovascular and overall mortality risks in association with higher dietary intakes of animal protein are, generally, largely in line with those from previous iso-caloric modeling studies in other prospective cohorts, although all studies varied with regard to the degree of macronutrient breakdown in statistical risk models, specific macronutrient substitutions considered (e.g., for vegetable protein or for carbohydrates) and additional covariates considered as confounding factors [5,6,7,8,9,10]. The studies included in the systematic review found that substituting animal protein for carbohydrate increased overall mortality risk, and substituting plant protein for animal protein or carbohydrate reduced overall mortality risk in minimally adjusted models. When the models were fully adjusted, the protective effect of plant protein intake attenuated but persisted, whereas the increased overall mortality risk associated with substituting animal protein for carbohydrate disappeared in some studies. In our fully adjusted analyses (adjusting for age, sex, total energy intake, BMI, smoking, alcohol consumption and dietary fiber intake), an increased mortality risk was found in association with higher intakes of animal protein as iso-caloric substitution for either carbohydrates or non-saturated fats, whereas a substitution for vegetable protein was associated with mortality risk only in models minimally adjusted for age, sex and total energy, somewhat in contrast to studies that had previously reported robust associations specifically between higher intakes of plant protein and reduced mortality risk. Furthermore, all studies, including ours, also indicated an association of animal vs. plant sources of dietary protein specifically with cardiovascular mortality. In the majority of studies, the evidence reported suggests that the associations of cardiovascular mortality risk with higher intakes of animal protein, or lower intakes of protein vegetal sources, may be driven by elevated consumption of red or processed meat, a further characteristic of diet for which increased risks of various chronic diseases and overall mortality are frequently also being reported [11,25]. It is important to note, however, that in our sensitivity analysis, further adjustment of red meat intake to our fully adjusted model did not significantly change the estimated higher cardiovascular mortality risk for the substitution of animal protein for mono- or di-saccharides and other complex carbohydrates, suggesting that the observed associations may be independent of, or at least not fully accounted for, by elevated consumption of red and processed meat. However, despite these and other heterogeneities between individual study reports, the overall picture emerging from our and previous studies indicates that a higher percent of dietary energy intake from animal protein, or a lower percent intake of non-animal protein from non-animal (plant) sources, is associated with an increased risk of overall mortality. 

The reviewed studies generally reported mortality hazards ratios only for selected macronutrient substitutions by animal or plant protein, but did not provide an overall picture of all possible pairwise substitutions between sub-types of protein, fat and carbohydrates. In our analysis, breaking down each macronutrient (protein, fat and carbohydrates) into their sub-types improved model fit, suggesting that improvement in model fit may not be entirely related to animal protein intake, but significant substitution of other macronutrients could have contributed to model fit. Significant substitution effects correspond to significant differences between weighted sums of positive and negative coefficients of nutrients considered, where this weighted difference is significantly different from zero. Therefore, not considering all pairwise macronutrient substitution could potentially limit the interpretation of strength and direction of associations as important, details might be lost when the macronutrients are aggregated versus broken down. Importantly, because we tested all possible pairwise substitutions, we found that—in addition to the association of animal protein with overall and cardiovascular mortality—poly-unsaturated fat substituted for saturated fat was associated with cancer mortality. 

The findings from our and previous iso-caloric modeling studies in prospective study cohorts stand in contrast with those from international correlation studies that have shown higher cancer incidence rates, but lower rates of overall mortality (with longer life expectancy), in countries that have higher per capita availability and percent energy intake from animal protein [4,29,30]. This stark contrast between findings from individual-level vs. aggregate-level data analyses suggests that findings from international correlation studies are very likely biased by unknown confounding or interaction factors that are associated with the “country” as a group variable, which may include differences in quality of housing, general availability of high-quality medical health care or other important general health determinants related to differences in economic development [30,31,32]. At the same time, it cannot be ruled out that the individual-level association between mortality risk and dietary intake of protein from animal vs. plant origin, as reported in our and other prospective cohort studies, is not itself also the subject of residual confounding, in spite of adjustments for other major risk factors.

All things considered, the robust associations seen between higher animal protein intake and overall and cardiovascular mortality in our study and those of others suggests that factors uniquely associated with animal protein could perhaps explain these associations. In past studies it was speculated that branched-chain amino acids, largely present in animal protein sources, could lead to insulin resistance and excess weight, which are important predictors of chronic disease incidence, particularly cardiovascular disease, and can ultimately increase mortality risk [33,34,35]. Another hypothesis is that other metabolic mechanisms, such as increased synthesis and biological activity of insulin-like-growth-factor (IGF)-1, have been postulated to link animal-protein-rich diets to increased anabolic activity and to cause the development of cancer cells [36,37]. While these and several other biological mechanisms have been theorized to link animal protein intake and higher mortality risks, a single clear biological pathway has yet to be identified. 

### Limitations and Strengths

A first limitation of this study is that our primary exposure, nutrient intake, was assessed at only one point in time, at the baseline recruitment. Therefore, there is a chance for the participants to have changed their dietary habits or lifestyle, which could result in attenuated associations. Second, nutrient intake information derived from self-administered FFQ inherently has considerable random measurement error, also leading to attenuation of rich associations. Third, the EPIC-Heidelberg cohort had a comparatively better socioeconomic indicator than the underlying population and, as a result, the nutrient intake measures may not entirely represent the underlying population. In terms of strength, this was a large prospective cohort study with detailed information collected about lifestyle habits such as smoking, alcohol consumption and BMI, which are strong confounders for the association of nutrient intake and mortality. Thus, we were able to adjust for all important known confounders of the association. That said, the possibility of residual confounding by unmeasured factors cannot be entirely eliminated.

## 5. Conclusions

Overall, the findings from our and other studies indicate that higher proportions of dietary energy from animal protein, combined with low energy intake from either carbohydrates or dietary fats, increases mortality risk. Crucially, animal protein was not the only macronutrient associated with increased mortality risk. Therefore, future studies should further attempt to establish a causal association—by addressing residual confounding issues in prospective cohort studies and by establishing clear biological mechanisms—between animal protein intake and overall and cause-specific mortality.

## Figures and Tables

**Table 1:** Sample characteristics.

Variables	Total (N = 24,106) n (%)/Mean (SD)	Alive (N = 20,077) n (%)/Mean (SD)	Dead (N = 4029) n (%)/Mean (SD)
Follow-up (years, median, inter-quartile range)	22.7 (21.7–23.8)	22.9 (22.1–23.9)	16.1 (10.6–22.5)
Cause of death			
Cancer			1603 (39.7)
Cardiovascular			982 (24.3)
Other causes			1444 (35.8)
Age at recruitment (years, median, inter-quartile range)	51.4 (43.5–57.5)	49.1 (42.8–56.2)	58.1 (52.6–62.1)
Age categories (years)			
<40	2333 (9.6)	2271 (11.3)	62 (1.5)
40–44	4685 (19.4)	4377 (21.8)	308 (7.6)
45–49	4274 (17.7)	3896 (19.4)	378 (9.3)
50–54	4486 (18.6)	3824 (19.1)	662 (16.4)
55–59	4268 (17.7)	3258 (16.2)	1010 (25.1)
≥60	4060 (16.8)	2451 (12.1)	1609 (39.9)
Sex			
Female	12,783 (53.1)	11,373 (56.6)	1410 (35)
Male	11,323 (46.9)	8704 (43.3)	2619 (65)
BMI (kg/m^2^)			
<18.5	201 (0.83)	171 (0.8)	30 (0.7)
≥18–<25	10,301 (42.7)	9183 (45.7)	1118 (27.7)
≥25–<30	9656 (40.1)	7823 (38.9)	1833 (45.5)
≥30–<35	3107 (12.8)	2319 (11.5)	788 (19.5)
≥35	841 (3.49)	581 (2.8)	260 (6.4)
Alcohol intake at recruitment (g/day)			
Non-drinkers	1300 (5.3)	924 (4.6)	376 (9.3)
0–6	5798 (24.1)	4936 (24.5)	862 (21.3)
6–12	6260 (25.9)	5474 (27.2)	786 (19.5)
12–24	4689 (19.4)	4007 (19.9)	682 (16.9)
24–60	4796 (19.9)	3896 (19.4)	900 (22.3)
60–96	1044 (4.3)	728 (3.6)	316 (7.8)
>96	219 (0.9)	112 (0.5)	107 (2.6)
Smoking status			
Never	10,217 (42.3)	8832 (43.9)	1385 (34.3)
Long-time quitter	5523 (22.9)	4635 (23.1)	888 (22.1)
Short-time quitter	2676 (11.1)	2259 (11.2)	417 (10.3)
Current light	2723 (11.3)	2246 (11.1)	477 (11.8)
Current heavy	2523 (10.4)	1767 (8.8)	756 (18.7)
Pipe/cigar/occasional	444 (1.84)	338 (1.6)	106 (2.6)
Total energy (kcal)	1973.6 (634.9)	1958.5 (621.2)	2049.2 (694.7)
Animal protein (kcal)	152.1 (69.4)	149.8 (67.6)	163.8 (76.9)
Non-animal protein (kcal)	139.6 (48.1)	139.1 (47.3)	142.2 (51.2)
Total fat (kcal)	687.7 (262.7)	685.1 (258.1)	700.5 (284.3)
Saturated fat (kcal)	285.8 (118.1)	285.5 (116.6)	287.5 (125.4)
Mono-unsaturated fat (kcal)	240.5 (96.3)	239.4 (94.5)	245.9 (104.6)
Poly-unsaturated fat (kcal)	112.9 (47.4)	112.1 (46.1)	117.6 (53.5)
Mono- and di-saccharide (kcal)	397.9 (200.3)	397.7 (196.2)	398.6 (220.1)
Other carbohydrate (kcal)	470.4 (170.1)	468.7 (168.3)	479.2 (178.2)

**Table 2:** Scenarios for the association of 3% substitution of energy among macronutrients with all-cause mortality (*n* = 24,106).

	Substitution of 3% of Energy from	Animal Protein (HR, 95% CI)	Non-Animal Protein (HR, 95% CI)	Saturated Fat (HR, 95% CI)	Mono-Unsaturated Fat (HR, 95% CI)	Poly-Unsaturated Fat (HR, 95% CI)	Mono- and Di-Saccharide (HR, 95% CI)	Other Carbohydrate (HR, 95% CI)
A	Animal protein for		1.12 (1.04–1.22) *	1.18 (1.12–1.25) *	1.12 (1.03–1.22) *	1.07 (1.01–1.15) *	1.12 (1.08–1.16) *	1.14 (1.11–1.19) *
	Non-animal protein for	0.88 (0.81–0.96) *		1.05 (0.96–1.14)	0.99 (0.89–1.11)	0.95 (0.86–1.05)	0.99 (0.91–1.08)	1.01 (0.93–1.11)
	Saturated fat for	0.84 (0.79–0.89) *	0.95 (0.87–1.03)		0.94 (0.84–1.05)	0.91 (0.85–0.96) *	0.94 (0.90–0.99) *	0.96 (0.91–1.01)
	Mono-unsaturated for	0.88 (0.81–0.96) *	1.003 (0.90–1.11)	1.05 (0.94–1.17)		0.95 (0.85–1.07)	0.99 (0.93–1.07)	1.02 (0.95–1.09)
	Poly-unsaturated for	0.92 (0.86–0.99) *	1.04 (0.94–1.15)	1.10 (1.04–1.16) *	1.04 (0.93–1.17)		1.04 (0.98–1.11)	1.06 (1.001–1.13) *
	Monosaccharide for	0.88 (0.85–0.92) *	1.004 (0.92–1.09)	1.05 (1.00–1.10) *	1.001 (0.93–1.07)	0.95 (0.90–1.01)		1.02 (1.001–1.04) *
	Other carbohydrate for	0.87 (0.84–0.90) *	0.98 (0.90–1.07)	1.03 (0.98–1.08)	0.98 (0.91–1.05)	0.93 (0.88–0.99) *	0.98 (0.96–0.99) *	
B	Animal protein for		1.07 (0.99–1.17)	1.11 (1.05–1.17) *	1.10 (1.01–1.20) *	1.01 (0.95–1.08)	1.07 (1.03–1.11) *	1.07 (1.03–1.11) *
	Non-animal protein for	0.92 (0.85–1.01)		1.03 (0.94–1.11)	1.02 (0.91–1.14)	0.94 (0.85–1.04)	0.99 (0.91–1.07)	0.99 (0.91–1.08)
	Saturated fat for	0.90 (0.85–0.95) *	0.97 (0.89–1.05)		0.99 (0.89–1.11)	0.91 (0.86–0.96) *	0.96 (0.91–1.01)	0.97 (0.92–1.02)
	Mono-unsaturated for	0.90 (0.83–0.98) *	0.97 (0.87–1.08)	1.01 (0.90–1.12)		0.91 (0.82–1.02)	0.97 (0.91–1.03)	0.97 (0.91–1.04)
	Poly-unsaturated for	0.98 (0.92–1.05)	1.06 (0.96–1.17)	1.09 (1.03–1.15) *	1.08 (0.97–1.21)		1.05 (0.99–1.12)	1.06 (0.99–1.12)
	Monosaccharide for	0.93 (0.90–0.96) *	1.01 (0.92–1.09)	1.03 (0.98–1.09)	1.03 (0.96–1.10)	0.94 (0.89–1.01)		1.01 (0.98–1.02)
	Other carbohydrate for	0.92 (0.89–0.96) *	1.001 (0.91–1.09)	1.03 (0.98–1.08)	1.02 (0.95–1.09)	0.94 (0.88–1.002)	0.99 (0.97–1.01)	
C	Animal protein for		1.02 (0.94–1.11)	1.09 (1.03–1.15) *	1.11 (1.02–1.21) *	0.98 (0.92–1.05)	1.06 (1.03–1.11) *	1.05 (1.01–1.09) *
	Non-animal protein for	0.97 (0.90–1.06)		1.07 (0.98–1.16)	1.09 (0.97–1.21)	0.96 (0.87–1.06)	1.04 (0.96–1.13)	1.03 (0.94–1.12)
	Saturated fat for	0.91 (0.86–0.96) *	0.93 (0.85–1.01)		1.01 (0.91–1.13)	0.90 (0.85–0.95) *	0.97 (0.92–1.02)	0.96 (0.91–1.01)
	Mono-unsaturated for	0.89 (0.82–0.97) *	0.91 (0.82–1.02)	0.98 (0.88–1.09)		0.88 (0.79–0.99)	0.95 (0.89–1.02)	0.94 (0.88–1.01)
	Poly-unsaturated for	1.01 (0.94–1.08)	1.03 (0.93–1.14)	1.10 (1.04–1.17) *	1.12 (1.01–1.26)		1.08 (1.01–1.14) *	1.06 (1.004–1.13) *
	Monosaccharide for	0.93 (0.90–0.97) *	0.95 (0.88–1.03)	1.02 (0.97–1.07)	1.04 (0.97–1.11)	0.92 (0.87–0.98) *		0.98 (0.96–1.01)
	Other carbohydrate for	0.94 (0.91–0.98) *	0.96 (0.88–1.05)	1.03 (0.98–1.09)	1.05 (0.98–1.13)	0.93 (0.88–0.99) *	1.01 (0.99–1.03)	

Note: * *p* < 0.05. Energy from alcohol and residual fat excluded from total energy calculations. Below and above the diagonal are reciprocal substitution effects. A: Adjusted for age, sex, total energy. B: Adjusted for age, sex, total energy, smoking, BMI. C: Adjusted for age, sex, total energy, smoking, BMI, alcohol intake at recruitment, fiber intake.

**Table 3:** List of recent past studies that examined the association of animal protein with all-cause mortality by substituting animal protein for other macronutrients in a healthy population at baseline.

	Author, Year	Model	N	Duration of Follow-Up (Years)	No. of Deaths	Adjusted Covariates	Cohort, Country	Nutrient/Food Breakdown	Interpretation of Results	Adjusted for Total Energy	Result *
1	Budhathoki et al., 2019 [8]	Nutrient density	70,696	18	All = 12,381 CVD = 3025 Cancer = 5055	Age, sex, BMI, smoking status, alcohol intake, physical activity, occupational status, coffee consumption and green tea consumption, total energy	Japan Public Health Center-based Prospective Cohort, Japan	Animal protein, plant protein, saturated fat, mono-unsaturated fat, polyunsaturated fat and other fat	Quintile categories	Yes	All-cause mortality Lower quintile of plant protein intake compared to highest quintile quintile 1, reference category quintile 2, 0.89 (0.83–0.95) quintile 3, 0.88 (0.82–0.95) quintile 4, 0.84 (0.77–0.92) quintile 5, 0.87 (0.78–0.96) Cardiovascular mortality Lower quintile of plant protein intake compared to highest quintile quintile 1, reference category quintile 5, 0.73 (0.59–0.91)
								Plant protein source, red meat, processed meat, chicken, egg, dairy, fish	3% energy substitution	Yes	All-cause mortality Plant protein for red meat = 0.66 (0.55–0.80) Plant protein for processed meat = 0.54 (0.38–0.75) Cardiovascular mortality Plant protein for red meat = 0.58 (0.39–0.86) Cancer mortality Plant protein for red meat = 0.61 (0.45–0.82) Plant protein for processed meat = 0.50 (0.30–0.85)
2	Chen et al., 2020 [6]	Nutrient density	7786	13	All = 3589 CVD = 877 Cancer = 896	Age, sex, study cohort, fiber, overall diet quality score, physical activity, education level, smoking status and BMI.	The Rotterdam Study (RS-I, RS-II and RS-III combined), the Netherlands	Animal protein, plant protein, saturated fatty acid, mono-unsaturated fatty acid, poly-unsaturated fatty acid, trans fat alcohol	5% energy substitution	Yes	All-cause mortality Animal protein for carbohydrate = 1.20 (1.05, 1.37) Cardiovascular mortality Animal protein for carbohydrate = 1.19 (1.04–1.37)
3	Huang et al., 2020 [10]	Nutrient density	416,104	16	All = 77,614 CVD = 22,228 Cancer = 28,099	Age, BMI, alcohol, smoking, physical activity, race or ethnic group, education level, marital status, diabetes, health status, vitamin supplement use, total energy, animal protein, saturated fat, poly-unsaturated fat, mono-unsaturated fat, trans fat, fiber, vegetable and fruits. For the endpoint with cancer, mortality model was further adjusted for history of cancer in a first-degree relative.	US National Institute of Health-AARP Diet and Health Study, the United States	Plant protein, animal protein, saturated fat, poly-unsaturated fat, monounsaturated fat, trans fat	Per 1 SD increase	Yes	All-cause mortality Per 1 SD increase in plant protein intake In men, 0.95 (0.94–0.97) In women, 0.95 (0.93–0.96) Cardiovascular mortality Per 1 SD increase in plant protein intake In men, 0.95 (0.93–0.98) In women, 0.93 (0.90–0.97)
								Red meat, white meat, dairy, egg	5% energy substitution	Yes	All-cause mortality Plant protein for red meat In men, 0.87 (0.85–0.90) In women, 0.85 (0.81–0.88) Plant protein for dairy In men, 0.92 (0.89–0.95) In women, 0.92 (0.89–0.95) Plant protein for egg In men, 0.76 (0.72–0.80) In women, 0.79 (0.73–0.85) Cardiovascular mortality Plant protein for red meat In men, 0.88 (0.83–0.93) In women, 0.82 (0.76–0.89) Plant protein for dairy In men, 0.89 (0.84–0.94) In women, 0.88 (0.82–0.95) Plant protein for egg In men, 0.74 (0.67–0.82) In women, 0.72 (0.63–0.83) Cancer mortality Plant protein for red meat In men, 0.93 (0.88–0.98) In women, 0.89 (0.83–0.95) Plant protein for egg In men, 0.85 (0.78–0.93) In women, 0.83 (0.73–0.93)
4	Kelemen et al., 2005 [25]	Nutrient density	29,017	15	All = 3978 CVD = 739 Cancer = 1676	Age, total energy, saturated fat, poly-unsaturated fat, mono-unsaturated fat, trans fat, total fiber, dietary cholesterol, dietary methionine, alcohol, smoking, activity level, BMI, history of hypertension, postmenopausal hormone use, multivitamin use, vitamin E supplement use, education and history of cancer	Iowa Women’s Health Study, the United States	Animal protein, plant protein, saturated fat, poly-unsaturated fat, monounsaturated fat and trans fat	Difference in median energy intake of protein between the highest and lowest quintile	Yes	Cardiovascular mortality Vegetable protein for carbohydrate = 0.70 (0.49–0.99) Vegetable protein for animal protein = 0.70 (0.51–0.98)
								Carbohydrate-rich food, legumes, dairy, eggs, red meats, poultry, fish	Servings per 1000 kcal	Yes	All-cause mortality Red meat for carbohydrate-rich food = 1.16 (1.02–1.32) Cardiovascular mortality Dairy for carbohydrate-rich food = 1.41 (1.07–1.87) Red meat for carbohydrate-rich food = 1.44 (1.06–1.94) Cancer mortality Legumes for carbohydrate-rich food = 1.23 (1.04–1.46)
5	Song et al., 2016 [9]	Nutrient density model	131,342	26	36,115	Age, multivitamin use, smoking status, pack-years of smoking, BMI, physical activity, alcohol consumption, hypertension, glycemic index, whole grains, fiber, fruits and vegetables.	The Nurses’ Health Study (NHS) and The Health Professionals Follow-up Study (HPFS), the United States	Plant protein, saturated, mono-unsaturated, poly-unsaturated and trans fatty acid.	10% energy substitution	Yes	Cardiovascular mortality Animal protein for carbohydrate = 1.08 (1.01–1.16)
6	Sun et al., 2021 [11]	Nutrient density model	102,521	18	All = 25,976 CVD = 6993 Cancer = 7516	Age, race/ethnicity, education, income, observational study/clinical trial, unopposed estrogen use, estrogen + progesterone use, smoking, physical activity, alcohol intake, total energy intake, baseline diabetes mellitus, baseline high cholesterol status, family history of heart attack/stroke, dietary fiber intake, glycemic load and BMI	Women’s Health Initiative, Unites States	Animal protein, plant protein, saturated fatty acids, poly-unsaturated fatty acid, mono-unsaturated fatty acids and trans fat	5% energy substitution	Yes	All-cause mortality plant protein for animal protein = 0.86 (0.81–0.91) Cardiovascular mortality plant protein for animal protein = 0.78 (0.70–0.87)
								Total red meat, unprocessed red meat, processed red meat, poultry, fish/shellfish, eggs, dairy products, legumes, nuts	OZ equivalent per day for red meat, poultry, fish, eggs, legumes and nuts Cup equivalent per day for dairy products	Yes	All-cause mortality Red meat for nuts = 0.89 (0.81–0.98) Eggs for nuts = 0.53 (0.45–0.61) Dairy for nuts = 0.88 (0.80–0.97) Legumes for nuts = 0.86 (0.74–0.99) Cardiovascular mortality Eggs for nuts = 0.44 (0.33–0.58) Dairy for nuts = 0.81 (0.67–0.97) Legumes for nuts = 0.70 (0.53–0.92) Cancer mortality Eggs for nuts = 0.59 (0.45–0.78)
7	Van den Brandt, 2019 [28]	Standard multivariate	13,823	10	All = 8823 CVD = 2985 Cancer = 3917	Age; cigarette smoking; number of cigarettes smoked per day; years of smoking; history of physician-diagnosed hypertension; diabetes; BMI; non-occupational physical activity; highest level of education; intake of alcohol, vegetables, and fruits; energy; use of nutritional supplement; and, in women, post-menopausal use of hormone replacement therapy	The Netherlands Cohort Study	Poultry, eggs, fish, nuts, pulses, low-fat dairy	50 g/day substitution	Yes	All-cause mortality Nuts for processed meat = 0.65 (0.49–0.85) Cardiovascular mortality Nuts for processed meat = 0.62 (0.44–0.88) Cancer mortality Nuts for processed meat = 0.73 (0.54–0.99)

* Only significant results shown. Complete results can be extracted from the cited studies in the reference list.

## Data Availability

Access to the data used for the analysis will be made available on request. Please contact the corresponding author.

## Supplementary Materials

The following supporting information can be downloaded at: https://www.mdpi.com/article/10.3390/nu15030794/s1, Table S1: Comparison of risk models for increasing breakdown of dietary energy source into macronutrient sub-types (likelihood ratio tests); Table S2: Search terms for systematic literature review, for studies relating animal protein intake to mortality risk, using iso-caloric modeling; Table S3: Estimated hazard ratios for cardiovascular mortality, in association with various pairwise macronutrient substitutions (substitution of 3% of energy intake); Table S4: Estimated hazard ratios for cancer mortality, in association with various pairwise macronutrient substitutions (substitution of 3% of energy intake); Table S5: Scenarios for the association of 3% substitution of energy among macronutrients with all-cause mortality, cardiovascular mortality and cancer mortality after removing participants with diabetes; Table S6: Scenarios for the association of 3% substitution of energy among macronutrients with all-cause mortality, cardiovascular mortality and cancer mortality after adding red meat intake to the maximally adjusted risk models; Table S7: Scenarios for the association of 3% substitution of energy among macronutrients with all-cause mortality, cardiovascular mortality and cancer mortality using standard multivariate model (“S” models).
