# Supplementary material for: Effect of Iso-Caloric Substitution of Animal Protein for Other Macro Nutrients on Risk of Overall, Cardiovascular and Cancer Mortality: Prospective Evaluation in EPIC-Heidelberg Cohort and Systematic Review
Source: Nutrients. 2023 Feb 3;15(3):794. doi: 10.3390/nu15030794 (PMC9920011; doi:10.3390/nu15030794)
Supplement: Supplementary file 1 [file nutrients-15-00794-s001.zip › Online supporting material_final.pdf]

Table S1. Comparison of risk models, for increasing breakdown of dietary energy sources into macronutrients sub-types (likelihood ratio tests).

	β-coefficients													
Model	Total protein	Animal protein	Non-animal protein	Total fat	Saturated fat	Mono-unsaturated fat	Poly-unsaturated fat	Total carbohydrate	Mono- and disaccharide	Other carbohydrate	Deviance (-2logL)	Chi-square	DOF	Likelihood ratio test (p-value)
1	0.12										71294.9			
2		0.12	0.03								71288.5	6.4	1	0.01
3		0.13	0.02	-0.01							71287.6	0.9	1	0.34
4		0.12	-0.01		-0.03	-0.01	0.05				71277.4	10.2	3	0.01
5		0.14	0.02		-0.02	0.01	0.07		0.02		71273.4	4	1	0.04

Note: '×' represents the macronutrients included in the model. All models were adjusted for total energy, age, and sex.

Legend: Model 1 is the simplest model where macronutrient is broken down into total protein and energy from other sources (i.e. total protein + energy from other sources = total energy intake). Every model that follows breaks down macronutrient into its sub-type (for example, in model 2, animal protein and non-animal protein is a breakdown of total protein). In the same way, each model (in descending order) is a sub-set of the previous model (for example: Model 3 is a sub-set of Model 2). Therefore, the log-likelihood ratio test estimates if breaking down macronutrients into its sub-types improves model fit significantly. In other words, we test if the coefficients of the sub-types of macronutrient are different from each other.

Supplementary note: In the model building step, we found that the model in which every macronutrient was broken down into its components had a better model fit compared to smaller nested models showing that the substitution effect (meaning 1 kcal of saturated fat has a significantly different coefficient than 1 kcal of poly unsaturated fat) of the sub-types of macronutrient may differ in the direction or magnitude of association. It is also important to note that the model with maximum macronutrient breakdown does not include one but 21 different pairwise substitution effect and we observed that all relevant substitutions were not to do with animal protein. We found that higher poly unsaturated fat (PUFAs) intake was associated with all-cause mortality (3% substitution of energy increased mortality risk by 6% to 11%) with similar strength as animal protein, particularly driven by the association of PUFAs with cancer mortality. From the model fit point of view, it should be noted that our analytical model that predicted higher mortality risk associated with animal protein intake had exactly same model fit as the one that predicted higher mortality risk associated with PUFAs intake. Because PUFAs have been recommended as a substitute for saturated fat, it is promoted in the public domain as "good" fat. Moreover, the current recommendation of substituting saturated fat with poly-unsaturated fat might be misleading because some PUFAs like Omega-6 has been linked with an increase in cancer risk. This findings show that when we examine the macronutrient and mortality association, it is prudent to estimate and/or report all possible macronutrient substitution effects so as to prevent important association from going unnoticed when focusing on one specific macronutrient substitution

Table S2. Search terms for systematic literature review, for studies relating animal protein intake to mortality risk, using iso-caloric modeling.

Date	Search term
From January 1, 2002 till October 12, 2022	((("dietary protein"[Title] OR "protein intake"[Title] OR "animal protein"[Title] OR "protein consumption"[Title] OR "animal proteins, dietary"[MeSH Terms] OR "Dietary Proteins"[MeSH Terms]) AND ("Mortality"[Title] OR "Death"[Title] OR "fatal"[Title] OR "Survival"[Title] OR "Mortality"[MeSH Subheading])))

Table S3. Estimated hazard ratios for cardiovascular mortality, in association with various pairwise macronutrient substitutions (substitution of 3% of energy intake) (n=24,106).

	**Substitution of 3% of energy from**	**Animal protein (HR, 95% CI)**	**Non-animal protein (HR, 95% CI)**	**Saturated Fat (HR, 95% CI)**	**Mono-unsaturated fat (HR, 95% CI)**	**Poly-unsaturated fat (HR, 95% CI)**	**Mono- and di-saccharide (HR, 95% CI)**	**other carbohydrate (HR, 95% CI)**
A	Animal protein for		1.08(0.92-1.27)	**1.23(1.11-1.38)***	**1.21(1.02-1.44)***	1.11(0.97-1.27)	**1.23(1.15-1.31)***	**1.24(1.16-1.34)***
	Non-animal protein for	0.92(0.78-1.08)		1.14(0.96-1.34)	1.12(0.90-1.39)	1.03(0.84-1.25)	1.13(0.96-1.33)	1.15(0.97-1.36)
	Saturated Fat for	**0.81(0.72-0.90)***	0.87(0.74-1.04)		0.98(0.79-1.22)	0.90(0.80-1.01)	0.99(0.89-1.10)	1.01(0.91-1.11)
	Mono-unsaturated for	**0.82(0.69-0.98)***	0.89(0.71-1.11)	1.02(0.81-1.26)		0.91(0.73-1.14)	1.01(0.88-1.16)	1.02(0.89-1.17)
	Poly-unsaturated for	0.90(0.78-1.03)	0.97(0.80-1.19)	1.11(0.99-1.25)	1.09(0.87-1.36)		1.11(0.98-1.24)	1.11(0.99-1.26)
	Monosaccharide for	**0.81(0.76-0.86)***	0.88(0.75-1.04)	1.01(0.90-1.12)	0.99(0.86-1.13)	0.90(0.80-1.02)		1.01(0.97-1.05)
	Other carbohydrate for	**0.80(0.74-0.86)***	0.86(0.73-1.03)	0.99(0.90-1.09)	0.98(0.85-1.12)	0.90(0.79-1.01)	0.99(0.95-1.03)	
B	Animal protein for		1.02(0.86-1.19)	**1.14(1.02-1.27)***	1.17(0.99-1.39)	1.04(0.91-1.18)	**1.15(1.07-1.23)***	**1.15(1.07-1.24)***
	Non-animal protein for	0.98(0.84-1.16)		1.11(0.94-1.31)	1.15(0.93-1.42)	1.02(0.84-1.24)	1.13(0.96-1.32)	1.13(0.95-1.34)
	Saturated Fat for	**0.87(0.78-0.98)***	0.90(0.76-1.06)		1.03(0.82-1.28)	0.91(0.81-1.02)	1.01(0.91-1.11)	1.01(0.91-1.12)
	Mono-unsaturated for	0.85(0.71-1.01)	0.86(0.70-1.07)	0.97(0.78-1.21)		0.88(0.71-1.11)	0.98(0.85-1.12)	0.98(0.85-1.12)
	Poly-unsaturated for	0.96(0.84-1.09)	0.98(0.80-1.19)	1.09(0.98-1.23)	1.13(0.90-1.40)		1.10(0.98-1.24)	1.11(0.98-1.25)
	Monosaccharide for	**0.86(0.81-0.93)***	0.88(0.75-1.04)	0.99(0.90-1.09)	1.02(0.89-1.17)	0.90(0.80-1.02)		1.002(0.99-1.003)
	Other carbohydrate for	**0.86(0.80-0.93)***	0.88(0.74-1.05)	0.99(0.89-1.09)	1.02(0.89-1.17)	0.90(0.80-1.02)	0.99(0.99-1.01)	
C	Animal protein for		0.98(0.83-1.16)	**1.13(1.01-1.26)***	1.18(0.99-1.39)	1.02(0.90-1.17)	**1.15(1.07-1.23)***	**1.13(1.05-1.22)***
	Non-animal protein for	1.02(0.86-1.20)		1.14(0.96-1.35)	1.19(0.96-1.48)	1.04(0.85-1.26)	1.16(0.99-1.37)	1.15(0.97-1.36)
	Saturated Fat for	**0.88(0.79-0.99)***	0.87(0.74-1.04)		1.04(0.83-1.29)	0.91(0.81-1.02)	1.01(0.91-1.12)	1.004(0.90-1.11)
	Mono-unsaturated for	0.84(0.71-1.01)	0.84(0.67-1.04)	0.96(0.77-1.20)		0.87(0.69-1.09)	0.97(0.85-1.11)	0.96(0.83-1.11)
	Poly-unsaturated for	0.98(0.85-1.11)	0.96(0.79-1.17)	1.09(0.98-1.23)	1.14(0.91-1.44)		1.11(0.99-1.26)	1.11(0.97-1.24)

Monosaccharide for	**0.86(0.81-0.93)***	0.86(0.72-1.01)	0.99(0.89-1.09)	1.03(0.90-1.17)	0.90(0.79-1.01)		0.98(0.94-1.03)
Other carbohydrate for	**0.88(0.81-0.95)***	0.86(0.73-1.03)	0.99(0.90-1.11)	1.04(0.90-1.20)	0.90(0.80-1.03)	1.02(0.97-1.06)	

Note: * $p < 0.05$

Energy from alcohol and residual fat excluded

Below and above the diagonal are reciprocal substitution effect

A: Adjusted for age, sex, total energy

B: Adjusted for age, sex, total energy, smoking, BMI

C: Adjusted for age, sex, total energy, smoking, BMI, alcohol intake at recruitment, fiber intake

Table S4. Estimated hazard ratios for cancer mortality, in association with various pairwise macronutrient substitutions (substitution of 3% of energy intake) (n=24,106).

	Substitution of 3% of energy from	Animal protein (HR, 95% CI)	Non-animal protein (HR, 95% CI)	Saturated Fat (HR, 95% CI)	Mono-unsaturated fat (HR, 95% CI)	Poly-unsaturated fat (HR, 95% CI)	Mono- and di-saccharide (HR, 95% CI)	other carbohydrate (HR, 95% CI)
A	Animal protein for		1.08(0.94-1.23)	**1.14(1.04-1.25)***	1.00(0.87-1.14)	1.04(0.93-1.16)	**1.06(1.01-1.12)***	**1.06(1.01-1.13)***
	Non-animal protein for	0.92(0.81-1.06)		1.05(0.92-1.21)	0.92(0.77-1.09)	0.96(0.81-1.13)	0.98(0.86-1.12)	0.98(0.85-1.13)
	Saturated Fat for	**0.87(0.80-0.96)***	0.95(0.82-1.08)		0.87(0.73-1.03)	**0.91(0.82-0.99)***	0.92(0.85-1.01)	0.93(0.86-1.01)
	Mono-unsaturated for	1.00(0.87-1.14)	1.08(0.91-1.29)	1.14(0.97-1.36)		1.04(0.87-1.24)	1.06(0.95-1.18)	1.06(0.95-1.19)
	Poly-unsaturated for	0.96(0.86-1.14)	1.06(0.88-1.23)	**1.09(1.01-1.21)***	0.96(0.80-1.14)		1.02(0.92-1.12)	1.02(0.92-1.13)
	Monosaccharide for	**0.94(0.89-0.99)***	1.02(0.89-1.16)	1.08(0.99-1.17)	0.94(0.84-1.05)	0.98(0.89-1.08)		1.01(0.97-1.03)
	Other carbohydrate for	**0.94(0.88-0.99)***	1.02(0.88-1.17)	1.07(0.99-1.16)	0.94(0.84-1.05)	0.98(0.88-1.08)	0.99(0.97-1.03)	
B	Animal protein for		1.04(0.91-1.19)	1.08(0.99-1.18)	0.99(0.86-1.13)	0.98(0.88-1.09)	1.02(0.96-1.08)	1.01(0.95-1.07)
	Non-animal protein for	0.96(0.84-1.09)		1.03(0.91-1.18)	0.94(0.79-1.12)	0.94(0.80-1.10)	0.97(0.85-1.11)	0.96(0.84-1.11)
	Saturated Fat for	0.92(0.84-1.01)	0.97(0.84-1.09)		0.91(0.76-1.08)	**0.91(0.82-0.99)***	0.94(0.87-1.02)	0.93(0.86-1.01)
	Mono-unsaturated for	1.01(0.88-1.16)	1.06(0.89-1.26)	1.09(0.92-1.31)		0.99(0.83-1.19)	1.03(0.93-1.15)	1.02(0.92-1.14)
	Poly-unsaturated for	1.02(0.91-1.13)	1.06(0.90-1.25)	**1.09(1.01-1.21)***	1.01(0.84-1.20)		1.03(0.94-1.14)	1.02(0.93-1.13)
	Monosaccharide for	0.98(0.92-1.04)	1.03(0.90-1.17)	1.06(0.98-1.14)	0.97(0.86-1.07)	0.97(0.87-1.06)		0.99(0.96-1.02)
	Other carbohydrate for	0.99(0.93-1.05)	1.04(0.90-1.19)	1.07(0.99-1.16)	0.98(0.87-1.08)	0.98(0.88-1.07)	1.01(0.98-1.04)	
C	Animal protein for		0.99(0.87-1.14)	1.06(0.97-1.16)	0.99(0.87-1.13)	0.95(0.85-1.06)	1.01(0.96-1.07)	0.99(0.93-1.05)
	Non-animal protein for	1.01(0.87-1.14)		1.07(0.93-1.22)	0.99(0.83-1.18)	0.95(0.81-1.12)	1.01(0.89-1.16)	0.99(0.87-1.14)
	Saturated Fat for	0.94(0.86-1.03)	0.93(0.81-1.07)		0.93(0.78-1.11)	**0.89(0.81-0.98)***	0.95(0.88-1.03)	0.93(0.86-1.01)

Mono-unsaturated for	1.01(0.88-1.14)	1.01(0.84-1.20)	1.07(0.90-1.28)		0.95(0.80-1.14)	1.02(0.91-1.13)	1.00(0.89-1.11)
Poly-unsaturated for	1.05(0.94-1.17)	1.05(0.89-1.23)	**1.12(1.02-1.23)***	1.05(0.87-1.25)		1.06(0.96-1.17)	1.04(0.94-1.15)
Monosaccharide for	0.99(0.93-1.04)	0.99(0.86-1.12)	1.05(0.97-1.13)	0.98(0.88-1.09)	0.94(0.85-1.04)		0.98(0.94-1.01)
Other carbohydrate for	1.01(0.95-1.07)	1.01(0.87-1.14)	1.07(0.99-1.16)	1.00(0.90-1.12)	0.96(0.86-1.06)	1.02(0.99-1.06)	

Note: * p < 0.05. Energy from alcohol and residual fat excluded. Below and above the diagonal are reciprocal substitution effect

A: Adjusted for age, sex, total energy

B: Adjusted for age, sex, total energy, smoking, BMI

C: Adjusted for age, sex, total energy, smoking, BMI, alcohol intake at recruitment, fiber intake

Table S5. Scenarios for the association of 3% substitution of energy among macronutrients with all-cause mortality, cardiovascular mortality, and cancer mortality after removing participants with diabetes (n=23,295). .

Overall mortality

	Substitution of 3% of energy from	Animal protein (HR, 95% CI)	Non-animal protein (HR, 95% CI)	Saturated Fat (HR, 95% CI)	Mono-unsaturated fat (HR, 95% CI)	Poly-unsaturated fat (HR, 95% CI)	Mono- and di- saccharide (HR, 95% CI)	other carbohydrate (HR, 95% CI)
A	Animal protein for		**1.14(1.04-1.24)***	**1.16(1.09-1.23)***	1.09(0.99-1.19)	1.07(0.99-1.15)	**1.09(1.05-1.14)***	**1.12(1.07-1.16)***
	Non-animal protein for	**0.87(0.80-0.95)***		1.01(0.93-1.11)	0.95(0.85-1.07)	0.93(0.84-1.04)	0.96(0.88-1.05)	0.98(0.89-1.08)
	Saturated Fat for	**0.86(0.81-0.91)***	0.98(0.89-1.07)		0.94(0.83-1.05)	**0.92(0.86-0.98)***	**0.94(0.89-0.99)***	0.96(0.91-1.01)
	Mono-unsaturated for	0.91(0.83-1.00)	1.04(0.93-1.17)	1.06(0.94-1.19)		0.98(0.86-1.11)	1.01(0.93-1.08)	1.02(0.95-1.11)
	Poly-unsaturated for	0.93(0.87-1.00)	1.06(0.95-1.18)	**1.08(1.02-1.15)***	1.02(0.90-1.15)		1.02(0.96-1.09)	1.04(0.98-1.12)
	Monosaccharide for	**0.91(0.87-0.94)***	1.03(0.95-1.13)	**1.05(1.003-1.11)***	0.99(0.92-1.06)	0.97(0.91-1.03)		**1.02(1.001-1.04)***
	Other carbohydrate for	**0.89(0.85-0.92)***	1.01(0.92-1.11)	1.03(0.98-1.09)	0.97(0.91-1.04)	0.95(0.89-1.02)	**0.98(0.96-0.99)***	
B	Animal protein for		1.08(0.99-1.18)	**1.08(1.02-1.15)**	1.07(0.98-1.17)	1.01(0.93-1.08)	**1.04(1.01-1.08)***	**1.05(1.01-1.09)***

	Non-animal protein for	0.92(0.84-1.01)		1.00(0.91-1.09)	0.99(0.88-1.11)	0.92(0.83-1.03)	0.96(0.88-1.05)	0.96(0.88-1.06)
	Saturated Fat for	**0.92(0.86-0.97)***	1.00(0.91-1.09)		0.99(0.88-1.11)	**0.92(0.87-0.98)***	0.96(0.91-1.01)	0.96(0.91-1.02)
	Mono-unsaturated for	0.92(0.84-1.01)	1.01(0.89-1.13)	1.01(0.89-1.13)		0.93(0.82-1.05)	0.97(0.91-1.01)	0.97(0.91-1.05)
	Poly-unsaturated for	0.99(0.92-1.06)	1.07(0.97-1.20)	**1.08(1.01-1.14)***	1.07(0.94-1.21)		1.04(0.97-1.11)	1.04(0.97-1.11)
	Monosaccharide for	**0.95(0.91-0.99)***	1.03(0.95-1.13)	1.03(0.98-1.09)	1.02(0.95-1.11)	0.96(0.90-1.02)		1.004(0.98-1.02)
	Other carbohydrate for	**0.95(0.91-0.98)***	1.03(0.94-1.13)	11.03(0.97-1.08)	1.02(0.95-1.10)	0.95(0.89-1.02)	0.99(0.97-1.01)	
C	Animal protein for		1.01(0.93-1.11)	**1.06(1.01-1.13)***	1.09(1.00-1.19)	0.96(0.90-1.04)	**1.04(1.004-1.08)***	1.02(0.98-1.06)
	Non-animal protein for	0.98(0.89-1.07)		1.04(0.95-1.14)	1.07(0.95-1.20)	0.95(0.85-1.05)	1.02(0.93-1.11)	1.01(0.91-1.10)
	Saturated Fat for	**0.93(0.88-0.99)***	0.95(0.87-1.04)		1.02(0.91-1.15)	**0.91(0.85-0.96)***	0.97(0.92-1.03)	0.96(0.91-1.01)
	Mono-unsaturated for	0.91(0.83-1.00)	0.93(0.83-1.04)	0.97(0.86-1.09)		0.88(0.78-1.00)	0.95(0.88-1.02)	0.93(0.87-1.01)
	Poly-unsaturated for	1.03(0.96-1.11)	1.05(0.94-1.16)	**1.10(1.03-1.17)***	1.12(1.00-1.27)		**1.07(1.01-1.14)***	1.05(0.99-1.12)
	Monosaccharide for	**0.96(0.92-0.99)***	0.97(0.89-1.06)	1.02(0.97-1.07)	1.04(0.97-1.12)	**0.93(0.87-0.99)***		0.98(0.96-1.004)
	Other carbohydrate for	0.97(0.93-1.01)	0.99(0.91-1.09)	1.04(0.98-1.10)	1.06(0.99-1.14)	0.94(0.88-1.01)	1.02(0.89-1.04)	

Cardiovascular mortality

	Substitution of 3% of energy from	Animal protein (HR, 95% CI)	Non-animal protein (HR, 95% CI)	Saturated Fat (HR, 95% CI)	Mono-unsaturated fat (HR, 95% CI)	Poly-unsaturated fat (HR, 95% CI)	Mono- and di- saccharide (HR, 95% CI)	other carbohydrate (HR, 95% CI)
A	Animal protein for		1.15(0.96-1.38)	**1.19(1.06-1.35)***	1.15(0.96-1.39)	1.10(0.95-1.27)	**1.18(1.09-1.27)***	**1.19(1.10-1.29)***
	Non-animal protein for	0.86(0.72-1.03)		1.03(0.86-1.24)	1.00(0.79-1.27)	0.95(0.76-1.19)	1.02(0.85-1.23)	1.03(0.85-1.25)

	Saturated Fat for	0.83(0.74-0.94)*	0.96(0.80-1.15)		0.96(0.76-1.23)	0.92(0.81-1.04)	0.98(0.88-1.10)	0.99(0.89-1.11)
	Mono-unsaturated for	0.86(0.71-1.04)	0.99(0.78-1.26)	1.03(0.81-1.31)		0.95(0.74-1.22)	1.02(0.88-1.18)	1.03(0.88-1.19)
	Poly-unsaturated for	0.90(0.78-1.04)	1.04(0.83-1.30)	1.08(0.95-1.23)	1.04(0.81-1.34)		1.07(0.94-1.22)	1.08(0.94-1.23)
	Monosaccharide for	0.84(0.78-0.91)*	0.97(0.81-1.16)	1.01(0.90-1.13)	0.97(0.84-1.13)	0.93(0.81-1.06)		1.01(0.96-1.05)
	Other carbohydrate for	0.83(0.77-0.90)*	0.96(0.79-1.17)	1.00(0.89-1.12)	0.97(0.83-1.13)	0.92(0.80-1.05)	0.99(0.95-1.03)	
B	Animal protein for		1.08(0.90-1.29)	1.11(0.98-1.24)	1.12(0.93-1.35)	1.03(0.89-1.19)	**1.11(1.03-1.19)***	**1.10(1.01-1.19)***
	Non-animal protein for	0.92(0.77-1.07)		1.02(0.85-1.22)	1.04(0.81-1.32)	0.95(0.76-1.18)	1.02(0.85-1.22)	1.02(0.84-1.23)
	Saturated Fat for	0.90(0.80-1.01)	0.97(0.81-1.17)		1.01(0.79-1.29)	0.93(0.81-1.05)	1.000(0.89-1.12)	0.99(0.89-1.11)
	Mono-unsaturated for	0.88(0.73-1.07)	0.96(0.75-1.22)	0.98(0.77-1.25)		0.91(0.71-1.17)	0.98(0.84-1.14)	0.98(0.84-1.14)
	Poly-unsaturated for	0.97(0.83-1.12)	1.05(0.84-1.30)	1.07(0.94-1.22)	1.09(0.85-1.40)		1.07(0.96-1.04)	1.07(0.93-1.22)
	Monosaccharide for	0.90(0.83-0.97)	0.97(0.81-1.16)	0.99(0.89-1.11)	1.01(0.87-1.17)	0.92(0.81-1.05)		0.99(0.95-1.03)
	Other carbohydrate for	0.90(0.83-0.98)	0.98(0.81-1.18)	1.00(0.89-1.11)	1.02(0.87-1.18)	0.93(0.81-1.06)	1.01(0.96-1.04)	
C	Animal protein for		1.02(0.85-1.23)	1.08(0.96-1.22)	1.14(0.95-1.37)	0.99(0.86-1.15)	**1.10(1.02-1.19)***	1.07(0.99-1.17)
	Non-animal protein for	0.97(0.81-1.17)		1.06(0.88-1.27)	1.11(0.87-1.41)	0.97(0.78-1.20)	1.07(0.89-1.29)	1.05(0.87-1.27)
	Saturated Fat for	0.91(0.81-1.03)	0.94(0.78-1.13)		1.05(0.82-1.33)	0.91(0.80-1.04)	1.01(0.90-1.13)	0.99(0.88-1.11)
	Mono-unsaturated for	0.87(0.72-1.05)	0.89(0.70-1.14)	0.95(0.74-1.21)		0.87(0.68-1.11)	0.96(0.83-1.12)	0.94(0.81-1.09)
	Poly-unsaturated for	1.00(0.86-1.16)	1.02(0.82-1.28)	1.09(0.96-1.24)	1.14(0.89-1.46)		1.10(0.97-1.26)	1.08(0.94-1.23)

	Monosaccharide for	**0.90(0.83-0.97)***	0.92(0.77-1.11)	0.98(0.88-1.10)	1.03(0.89-1.20)	0.90(0.79-1.02)		0.97(0.93-1.02)
	Other carbohydrate for	0.92(0.85-1.00)	0.95(0.78-1.14)	1.01(0.90-1.13)	1.06(0.91-1.23)	0.92(0.81-1.05)	1.02(0.98-1.07)	
	**Cancer mortality**							
	Substitution of 3% of energy from	Animal protein (HR, 95% CI)	Non-animal protein (HR, 95% CI)	Saturated Fat (HR, 95% CI)	Mono-unsaturated fat (HR, 95% CI)	Poly-unsaturated fat (HR, 95% CI)	Mono- and di- saccharide (HR, 95% CI)	other carbohydrate (HR, 95% CI)
A	Animal protein for		1.06(0.92-1.22)	**1.14(1.04-1.24)***	1.01(0.88-1.16)	1.04(0.93-1.16)	**1.06(1.01-1.13)***	**1.07(1.01-1.14)***
	Non-animal protein for	0.94(0.82-1.08)		1.07(0.93-1.23)	0.95(0.79-1.13)	0.97(0.82-1.15)	1.01(0.87-1.15)	1.01(0.87-1.17)
	Saturated Fat for	**0.87(0.80-0.96)***	0.93(0.81-1.07)		0.88(0.74-1.06)	0.91(0.82-1.01)	0.93(0.86-1.01)	0.94(0.86-1.02)
	Mono-unsaturated for	0.98(0.85-1.13)	1.05(0.87-1.25)	1.12(0.94-1.34)		1.02(0.85-1.24)	1.05(0.94-1.18)	1.06(0.95-1.19)
	Poly-unsaturated for	0.96(0.85-1.07)	1.02(0.86-1.20)	1.09(0.99-1.20)	0.97(0.80-1.17)		1.02(0.92-1.13)	1.03(0.93-1.14)
	Monosaccharide for	**0.93(0.88-0.99)***	0.99(0.86-1.21)	1.06(0.98-1.15)	0.94(0.84-1.06)	0.97(0.88-1.07)		1.01(0.97-1.03)
	Other carbohydrate for	**0.92(0.87-0.98)***	0.98(0.85-1.14)	1.05(0.97-1.15)	0.94(0.84-1.05)	0.96(0.87-1.07)	0.99(0.96-1.02)	
B	Animal protein for		1.02(0.89-1.17)	1.08(0.98-1.18)	1.01(0.87-1.15)	0.98(0.88-1.10)	1.03(0.97-1.09)	1.02(0.96-1.08)
	Non-animal protein for	0.97(0.84-1.11)		1.05(0.91-1.20)	0.97(0.81-1.17)	0.95(0.81-1.13)	1.003(0.87-1.14)	0.99(0.86-1.14)
	Saturated Fat for	0.92(0.84-1.01)	0.95(0.82-1.09)		0.93(0.77-1.11)	0.91(0.82-1.00)	0.95(0.87-1.03)	0.94(0.87-1.02)
	Mono-unsaturated for	0.99(0.86-1.14)	1.02(0.85-1.22)	1.07(0.89-1.28)		0.97(0.81-1.18)	1.02(0.91-1.14)	1.01(0.91-1.13)
	Poly-unsaturated for	1.01(0.90-1.13)	1.04(0.88-1.23)	1.09(0.99-1.20)	1.02(0.84-1.23)		1.04(0.94-1.15)	1.03(0.93-1.15)
	Monosaccharide for	0.98(0.91-1.02)	0.99(0.87-1.14)	1.04(0.96-1.13)	0.97(0.87-1.09)	0.95(0.86-1.05)		0.99(0.96-1.02)

	Other carbohydrate for	0.97(0.91-1.04)	1.01(0.87-1.15)	1.05(0.97-1.14)	0.98(0.87-1.10)	0.96(0.86-1.06)	1.01(0.97-1.03)	
C	Animal protein for		0.97(0.84-1.12)	1.06(0.96-1.16)	1.01(0.88-1.16)	0.94(0.84-1.06)	1.02(0.96-1.08)	0.99(0.93-1.06)
	Non-animal protein for	1.02(0.89-1.17)		1.08(0.94-1.25)	1.04(0.86-1.24)	0.97(0.82-1.14)	1.04(0.91-1.19)	1.02(0.88-1.18)
	Saturated Fat for	0.94(0.86-1.03)	0.92(0.80-1.05)		0.95(0.79-1.14)	**0.89(0.81-0.98)***	0.96(0.88-1.04)	0.94(0.86-1.02)
	Mono-unsaturated for	0.98(0.85-1.13)	0.96(0.80-1.15)	1.04(0.87-1.25)		0.93(0.77-1.13)	1.01(0.89-1.12)	0.98(0.87-1.11)
	Poly-unsaturated for	1.05(0.94-1.17)	1.02(0.87-1.21)	**1.11(1.01-1.23)***	1.06(0.88-1.29)		1.07(0.97-1.18)	1.05(0.94-1.16)
	Monosaccharide for	0.98(0.92-1.03)	0.95(0.83-1.09)	1.03(0.95-1.12)	0.99(0.88-1.11)	0.93(0.84-1.02)		0.97(0.94-1.01)
	Other carbohydrate for	1.00(0.93-1.06)	0.97(0.84-1.12)	1.06(0.97-1.15)	1.01(0.91-1.13)	0.95(0.85-1.05)	1.02(0.98-1.05)	

Note: * $p < 0.05$. Energy from alcohol and residual fat excluded. Below and above the diagonal are reciprocal substitution effect

A: Adjusted for age, sex, total energy

B: Adjusted for age, sex, total energy, smoking, BMI

C: Adjusted for age, sex, total energy, smoking, BMI, alcohol intake at recruitment, fiber intake

Table S6. Scenarios for the association of 3% substitution of energy among macronutrients with all-cause mortality, cardiovascular mortality, and cancer mortality after adding red meat intake to the maximally adjusted risk models.

Substitution of 3% of energy from	Animal protein (HR, 95% CI)	Non-animal protein (HR, 95% CI)	Saturated Fat (HR, 95% CI)	Mono-unsaturated fat (HR, 95% CI)	Poly-unsaturated fat (HR, 95% CI)	Mono- and di- saccharide (HR, 95% CI)	other carbohydrate (HR, 95% CI)
**Overall mortality**							
Animal protein for		1.02(0.94-1.11)	**1.10(1.03-1.17)***	**1.11(1.02-1.21)***	0.99(0.92-1.06)	**1.07(1.02-1.11)***	**1.06(1.01-1.11)***
Non-animal protein for	0.97(0.89-1.06)		1.07(0.98-1.16)	1.09(0.97-1.21)	0.96(0.87-1.06)	1.04(0.96-1.13)	1.03(0.94-1.12)
Saturated Fat for	**0.90(0.85-0.96)***	0.93(0.85-1.01)		1.01(0.91-1.13)	**0.90(0.85-0.95)***	0.97(0.92-1.02)	0.96(0.91-1.01)
Mono-unsaturated for	**0.89(0.82-0.97)***	0.91(0.82-1.02)	0.98(0.88-1.09)		**0.88(0.79-0.99)***	0.95(0.89-1.02)	0.94(0.88-1.01)
Poly-unsaturated for	1.00(0.93-1.08)	1.03(0.93-1.14)	**1.10(1.04-1.17)***	**1.12(1.00-1.26)***		**1.08(1.01-1.14)***	**1.06(1.00-1.13)***
Monosaccharide for	**0.93(0.89-0.97)***	0.95(0.88-1.03)	1.02(0.97-1.08)	1.04(0.97-1.11)	**0.92(0.87-0.98)***		0.98(0.96-1.00)
Other carbohydrate for	**0.94(0.90-0.98)***	0.96(0.89-1.05)	1.03(0.98-1.09)	1.05(0.98-1.13)	**0.93(0.88-0.99)***	1.01(0.99-1.03)	
**Cardiovascular mortality**							
Animal protein for		0.97(0.81-1.15)	1.10(0.98-1.25)	1.16(0.97-1.38)	1.01(0.87-1.16)	**1.13(1.04-1.22)***	**1.11(1.02-1.21)***
Non-animal protein for	1.02(0.86-1.22)		1.14(0.96-1.35)	1.19(0.96-1.48)	1.04(0.85-1.26)	1.16(0.98-1.36)	1.15(0.97-1.36)
Saturated Fat for	0.90(0.79-1.01)	0.87(0.73-1.03)		1.04(0.83-1.30)	0.91(0.80-1.02)	1.01(0.91-1.12)	1.00(0.90-1.12)
Mono-unsaturated for	0.86(0.72-1.02)	0.83(0.67-1.03)	0.95(0.76-1.19)		0.87(0.69-1.09)	0.97(0.85-1.11)	0.96(0.83-1.10)
Poly-unsaturated for	0.98(0.85-1.13)	0.96(0.79-1.16)	1.09(0.97-1.23)	1.14(0.91-1.43)		1.11(0.99-1.25)	1.10(0.97-1.25)
Monosaccharide for	**0.88(0.81-0.96)***	0.86(0.73-1.01)	0.98(0.88-1.08)	1.02(0.89-1.17)	0.89(0.79-1.00)		0.99(0.94-1.03)
Other carbohydrate for	**0.89(0.82-0.97)***	0.86(0.73-1.02)	0.99(0.89-1.10)	1.03(0.90-1.19)	0.90(0.80-1.02)	1.01(0.96-1.05)	
**Cancer mortality**							
Animal protein for		0.98(0.85-1.13)	1.05(0.95-1.16)	0.98(0.85-1.13)	0.94(0.84-1.06)	1.00(0.94-1.07)	0.98(0.91-1.05)
Non-animal protein for	1.01(0.87-1.16)		1.06(0.93-1.22)	0.99(0.84-1.18)	0.95(0.81-1.12)	1.01(0.89-1.16)	0.99(0.86-1.14)
Saturated Fat for	0.94(0.85-1.04)	0.93(0.81-1.07)		0.93(0.78-1.11)	**0.89(0.81-0.98)***	0.95(0.88-1.03)	0.93(0.86-1.01)
Mono-unsaturated for	1.01(0.88-1.16)	1.00(0.84-1.19)	1.06(0.89-1.27)		0.95(0.80-1.14)	1.02(0.91-1.13)	1.00(0.89-1.11)
Poly-unsaturated for	1.05(0.94-1.18)	1.04(0.89-1.22)	**1.11(1.01-1.22)***	1.04(0.87-1.25)		1.06(0.96-1.17)	1.04(0.94-1.15)
Monosaccharide for	0.99(0.92-1.06)	0.98(0.86-1.12)	1.04(0.96-1.13)	0.98(0.88-1.09)	0.94(0.85-1.03)		0.98(0.94-1.01)
Other carbohydrate for	1.01(0.94-1.08)	1.00(0.87-1.15)	1.06(0.98-1.16)	1.00(0.89-1.11)	0.95(0.86-1.05)	1.02(0.98-1.05)	

Note: * $p < 0.05$. Energy from alcohol and residual fat excluded. Below and above the diagonal are reciprocal substitution effect. Adjusted for age, sex, total energy, smoking, BMI, alcohol intake at recruitment, fiber intake, and red meat intake

Table S7. Scenarios for the association of 3% substitution of energy among macronutrients with all-cause mortality, cardiovascular mortality, and cancer mortality using standard multivariate model ("S" models).

Overall mortality

	**Substitution of 3% of energy from**	**Animal protein (HR, 95% CI)**	**Non-animal protein (HR, 95% CI)**	**Saturated Fat (HR, 95% CI)**	**Mono-unsaturated fat (HR, 95% CI)**	**Poly-unsaturated fat (HR, 95% CI)**	**Mono- and di- saccharide (HR, 95% CI)**	**other carbohydrate (HR, 95% CI)**
A	Animal protein for		**1.12(1.02-1.21)***	**1.17(1.11-1.24)***	**1.12(1.02-1.22)***	1.05(0.97-1.12)	**1.10(1.07-1.14)***	**1.14(1.10-1.19)***
	Non-animal protein for	**0.88(0.81-0.96)***		1.04(0.96-1.14)	1.00(0.89-1.12)	0.93(0.84-1.04)	0.99(0.90-1.07)	1.02(0.93-1.12)
	Saturated Fat for	**0.84(0.79-0.89)***	0.95(0.87-1.04)		0.95(0.85-1.06)	**0.89(0.84-0.95)***	**0.94(0.89-0.99)***	0.97(0.92-1.02)
	Mono-unsaturated for	**0.88(0.81-0.96)***	0.99(0.89-1.11)	1.04(0.93-1.17)		0.93(0.83-1.05)	0.98(0.92-1.06)	1.02(0.95-1.09)
	Poly-unsaturated for	**0.92(0.86-0.99)***	1.06(0.95-1.18)	**1.11(1.05-1.18)***	1.06(0.94-1.20)		1.05(0.99-1.12)	**1.09(1.02-1.16)***
	Monosaccharide for	**0.88(0.85-0.92)***	1.01(0.92-1.10)	**1.06(1.00-1.11)***	1.01(0.94-1.08)	0.94(0.89-1.01)		**1.03(1.01-1.05)***
	Other carbohydrate for	**0.87(0.84-0.90)***	0.97(0.89-1.06)	1.02(0.97-1.08)	0.97(0.91-1.05)	**0.91(0.85-0.97)***	**0.96(0.94-0.98)***	
B	Animal protein for		1.07(0.98-1.16)	**1.09(1.03-1.16)***	**1.10(1.01-1.20)***	0.99(0.92-1.06)	**1.05(1.02-1.09)**	**1.07(1.03-1.11)***
	Non-animal protein for	0.92(0.85-1.00)		1.02(0.93-1.11)	1.02(0.92-1.15)	0.92(0.83-1.02)	0.98(0.90-1.07)	1.00(0.91-1.09)
	Saturated Fat for	**0.90(0.85-0.95)***	0.97(0.89-1.06)		1.01(0.89-1.12)	**0.90(0.85-0.95)***	0.96(0.91-1.01)	0.97(0.92-1.03)
	Mono-unsaturated for	**0.90(0.83-0.98)***	0.97(0.86-1.08)	0.99(0.88-1.11)		0.89(0.79-1.01)	0.95(0.89-1.02)	0.97(0.90-1.04)
	Poly-unsaturated for	0.98(0.92-1.05)	1.08(0.97-1.20)	**1.10(1.04-1.17)***	1.11(0.99-1.25)		**1.06(1.00-1.13)***	**1.08(1.01-1.15)***
	Monosaccharide for	**0.93(0.90-0.96)***	1.01(0.93-1.10)	1.03(0.98-1.09)	1.04(0.97-1.11)	**0.93(0.87-0.99)***		1.01(0.99-1.03)
	Other carbohydrate for	**0.92(0.89-0.96)***	1.00(0.91-1.09)	1.02(0.97-1.07)	1.02(0.95-1.10)	**0.92(0.86-0.98)***	0.98(0.96-1.00)	

C	Animal protein for		1.01(0.93-1.10)	**1.07(1.01-1.14)***	**1.12(1.03-1.22)***	0.95(0.89-1.02)	**1.05(1.01-1.09)***	**1.04(1.01-1.08)***
	Non-animal protein for	0.97(0.90-1.06)		1.06(0.97-1.16)	1.10(0.98-1.23)	0.94(0.85-1.04)	1.04(0.95-1.13)	1.03(0.94-1.12)
	Saturated Fat for	**0.91(0.86-0.96)***	0.94(0.86-1.02)		1.04(0.92-1.16)	**0.88(0.83-0.94)***	0.97(0.92-1.03)	0.97(0.92-1.02)
	Mono-unsaturated for	**0.89(0.82-0.97)***	0.90(0.80-1.01)	0.96(0.86-1.07)		**0.85(0.75-0.96)***	0.94(0.87-1.00)	0.93(0.87-1.00)
	Poly-unsaturated for	1.01(0.94-1.08)	1.05(0.95-1.17)	**1.12(1.05-1.19)***	**1.17(1.04-1.31)***		**1.10(1.03-1.17)***	**1.09(1.02-1.16)***
	Monosaccharide for	**0.93(0.90-0.97)***	0.96(0.88-1.04)	1.02(0.97-1.07)	1.06(0.99-1.13)	**0.90(0.85-0.96)***		0.99(0.97-1.01)
	Other carbohydrate for	**0.94(0.91-0.98)***	0.96(0.88-1.05)	1.02(0.97-1.08)	1.07(0.99-1.14)	**0.91(0.85-0.97)***	1.00(0.98-1.02)	
	**Cardiovascular mortality**							
	Substitution of 3% of energy from	Animal protein (HR, 95% CI)	Non-animal protein (HR, 95% CI)	Saturated Fat (HR, 95% CI)	Mono-unsaturated fat (HR, 95% CI)	Poly-unsaturated fat (HR, 95% CI)	Mono- and di- saccharide (HR, 95% CI)	other carbohydrate (HR, 95% CI)
A	Animal protein for		1.02(0.87-1.21)	**1.23(1.10-1.38)***	**1.23(1.04-1.46)***	1.08(0.94-1.23)	**1.22(1.14-1.31)***	**1.26(1.17-1.36)***
	Non-animal protein for	0.92(0.78-1.08)		**1.20(1.01-1.42)***	1.20(0.96-1.50)	1.05(0.85-1.29)	1.19(1.00-1.41)	**1.23(1.03-1.46)***
	Saturated Fat for	**0.80(0.72-0.90)***	**0.83(0.70-0.98)***		0.99(0.79-1.25)	0.87(0.77-0.98)	0.99(0.88-1.10)	1.02(0.91-1.13)
	Mono-unsaturated for	**0.82(0.69-0.97)***	0.83(0.66-1.03)	1.00(0.79-1.25)		0.87(0.69-1.10)	0.99(0.86-1.14)	1.02(0.88-1.17)
	Poly-unsaturated for	0.89(0.78-1.01)	0.95(0.77-1.17)	**1.14(1.01-1.29)***	1.14(0.90-1.44)		1.13(1.00-1.28)	**1.17(1.02-1.33)***
	Monosaccharide for	**0.81(0.75-0.86)***	**0.83(0.70-0.99)***	1.00(0.90-1.12)	1.00(0.87-1.15)	0.88(0.77-0.99)		1.03(0.99-1.07)
	Other carbohydrate for	**0.80(0.74-0.86)***	**0.81(0.68-0.97)***	0.97(0.88-1.09)	0.97(0.84-1.12)	0.85(0.75-0.97)	0.97(0.93-1.01)	
B	Animal protein for		0.97(0.82-1.15)	**1.14(1.01-1.28)***	**1.20(1.01-1.42)***	1.01(0.88-1.16)	**1.15(1.07-1.23)***	**1.16(1.08-1.25)***

	Non-animal protein for	0.97(0.83-1.15)		1.17(0.99-1.38)	1.23(0.98-1.53)	1.03(0.84-1.27)	**1.18(1.00-1.39)**	**1.19(1.00-1.42)***
	Saturated Fat for	**0.87(0.78-0.97)***	0.85(0.72-1.01)		1.05(0.83-1.31)	0.88(0.78-0.99)	1.00(0.90-1.12)	1.02(0.91-1.13)
	Mono-unsaturated for	**0.85(0.71-1.00)***	0.81(0.65-1.01)	0.95(0.75-1.19)		0.84(0.66-1.06)	0.96(0.83-1.10)	0.97(0.84-1.11)
	Poly-unsaturated for	0.95(0.84-1.09)	0.96(0.78-1.18)	1.12(1.00-1.27)	1.18(0.93-1.49)		**1.13(1.00-1.28)**	**1.15(1.01-1.31)***
	Monosaccharide for	**0.86(0.80-0.93)***	**0.84(0.71-1.00)**	0.99(0.89-1.10)	1.04(0.90-1.19)	0.87(0.77-0.99)		1.01(0.97-1.05)
	Other carbohydrate for	**0.86(0.80-0.93)***	**0.83(0.70-0.99)***	0.98(0.88-1.09)	1.02(0.89-1.18)	0.86(0.76-0.98)	0.98(0.95-1.02)	
C	Animal protein for		0.93(0.79-1.10)	**1.13(1.01-1.27)***	**1.21(1.02-1.43)***	0.99(0.86-1.13)	**1.15(1.07-1.23)***	**1.14(1.05-1.23)***
	Non-animal protein for	1.01(0.85-1.19)		**1.21(1.02-1.43)***	**1.29(1.03-1.61)***	1.05(0.86-1.29)	**1.22(1.03-1.45)***	**1.21(1.02-1.45)***
	Saturated Fat for	**0.88(0.78-0.98)***	**0.82(0.69-0.98)***		1.06(0.85-1.33)	**0.87(0.77-0.98)***	1.01(0.91-1.12)	1.00(0.90-1.12)
	Mono-unsaturated for	**0.84(0.71-1.00)***	**0.77(0.61-0.96)***	0.93(0.74-1.17)		0.81(0.64-1.03)	0.95(0.82-1.09)	0.94(0.82-1.08)
	Poly-unsaturated for	0.97(0.84-1.10)	0.94(0.77-1.15)	**1.14(1.01-1.29)***	1.22(0.96-1.54)		**1.16(1.02-1.31)***	**1.15(1.01-1.30)***
	Monosaccharide for	**0.86(0.81-0.93)***	**0.81(0.68-0.96)***	0.98(0.88-1.09)	1.05(0.91-1.20)	**0.86(0.76-0.97)***		0.99(0.95-1.03)
	Other carbohydrate for	**0.87(0.81-0.94)***	**0.82(0.68-0.97)***	0.99(0.88-1.10)	1.05(0.92-1.21)	**0.86(0.76-0.98)***	1.00(0.96-1.05)	

Cancer mortality

	Substitution of 3% of energy from	Animal protein (HR, 95% CI)	Non-animal protein (HR, 95% CI)	Saturated Fat (HR, 95% CI)	Mono-unsaturated fat (HR, 95% CI)	Poly-unsaturated fat (HR, 95% CI)	Mono- and di- saccharide (HR, 95% CI)	other carbohydrate (HR, 95% CI)
A	Animal protein for		1.10(0.96-1.27)	**1.15(1.05-1.25)***	1.00(0.87-1.14)	1.03(0.92-1.15)	1.05(0.99-1.11)	**1.07(1.01-1.14)***
	Non-animal protein for	0.92(0.80-1.05)		1.03(0.90-1.19)	0.90(0.75-1.08)	0.93(0.78-1.10)	0.95(0.82-1.09)	0.96(0.83-1.12)

	Saturated Fat for	**0.87(0.80-0.95)***	0.96(0.83-1.10)		0.87(0.72-1.04)	**0.89(0.81-0.98)***	**0.91(0.84-0.99)***	0.93(0.85-1.01)
	Mono-unsaturated for	1.00(0.87-1.14)	1.10(0.92-1.32)	1.15(0.96-1.37)		1.03(0.85-1.24)	1.05(0.94-1.17)	1.07(0.95-1.20)
	Poly-unsaturated for	0.96(0.86-1.06)	1.07(0.90-1.27)	1.11(1.01-1.22)	0.97(0.80-1.17)		1.02(0.92-1.13)	1.04(0.93-1.15)
	Monosaccharide for	**0.94(0.88-0.99)***	1.05(0.91-1.20)	1.09(1.00-1.18)	0.94(0.84-1.06)	0.97(0.88-1.08)		1.01(0.98-1.04)
	Other carbohydrate for	**0.93(0.88-0.99)***	1.03(0.89-1.19)	1.07(0.98-1.16)	0.93(0.83-1.04)	0.96(0.86-1.07)	0.98(0.95-1.01)	
B	Animal protein for		1.07(0.94-1.23)	1.08(0.99-1.19)	0.99(0.87-1.14)	0.97(0.87-1.09)	1.02(0.96-1.08)	1.01(0.95-1.08)
	Non-animal protein for	0.95(0.83-1.08)		1.01(0.88-1.16)	0.92(0.77-1.10)	0.90(0.76-1.07)	0.94(0.82-1.08)	0.94(0.81-1.09)
	Saturated Fat for	0.92(0.84-1.00)	0.99(0.86-1.13)		0.91(0.76-1.09)	**0.89(0.81-0.98)***	0.93(0.86-1.01)	0.93(0.86-1.01)
	Mono-unsaturated for	1.01(0.88-1.15)	1.07(0.90-1.29)	1.09(0.91-1.30)		0.98(0.81-1.18)	1.02(0.91-1.14)	1.02(0.91-1.13)
	Poly-unsaturated for	1.01(0.91-1.12)	1.10(0.93-1.30)	**1.11(1.01-1.22)***	1.02(0.84-1.23)		1.04(0.94-1.15)	1.04(0.93-1.15)
	Monosaccharide for	0.97(0.92-1.03)	1.05(0.92-1.20)	1.06(0.98-1.15)	0.97(0.87-1.09)	0.95(0.86-1.06)		0.99(0.96-1.02)
	Other carbohydrate for	0.98(0.92-1.04)	1.05(0.91-1.22)	1.06(0.98-1.16)	0.98(0.87-1.09)	0.96(0.86-1.06)	1.00(0.97-1.03)	
C	Animal protein for		1.02(0.89-1.17)	1.06(0.97-1.16)	1.01(0.88-1.15)	0.94(0.84-1.05)	1.01(0.95-1.07)	0.99(0.93-1.06)
	Non-animal protein for	1.00(0.87-1.14)		1.04(0.90-1.19)	0.98(0.82-1.18)	0.92(0.77-1.08)	0.98(0.86-1.13)	0.97(0.84-1.12)
	Saturated Fat for	0.93(0.85-1.02)	0.96(0.83-1.10)		0.95(0.79-1.13)	**0.88(0.80-0.97)***	0.95(0.87-1.03)	0.93(0.86-1.02)
	Mono-unsaturated for	1.00(0.87-1.15)	1.01(0.84-1.21)	1.05(0.88-1.25)		0.93(0.77-1.12)	1.00(0.89-1.11)	0.98(0.88-1.10)
	Poly-unsaturated for	1.04(0.94-1.16)	1.08(0.91-1.28)	**1.13(1.02-1.24)***	1.07(0.88-1.29)		1.07(0.97-1.18)	1.05(0.95-1.17)

Monosaccharide for	0.98(0.93-1.04)	1.01(0.88-1.16)	1.05(0.96-1.14)	0.99(0.89-1.11)	0.93(0.84-1.03)		0.98(0.95-1.02)
Other carbohydrate for	1.00(0.94-1.06)	1.02(0.88-1.18)	1.06(0.97-1.16)	1.01(0.90-1.13)	0.94(0.85-1.04)	1.01(0.98-1.04)	

Note: * $p < 0.05$. Energy from alcohol and residual fat excluded. Below and above the diagonal are reciprocal substitution effect

A: Adjusted for age, sex, total energy

B: Adjusted for age, sex, total energy, smoking, BMI

C: Adjusted for age, sex, total energy, smoking, BMI, alcohol intake at recruitment, fiber intake
